# Splanchnectopia Accompanied With Atrial Septal Defect: A Case Report

**DOI:** 10.4021/cr20e

**Published:** 2011-05-20

**Authors:** Ning Bao, Yu Zhang

**Affiliations:** a Medical undergraduate, Jilin University, Jilin province, China; b Department of Cardiology, the First Hospital of Jilin University, Changchun 130021, Jilin province, China

**Keywords:** Splanchnectopia, Atrial septal defect, Pneumonia, Misdiagnosis

## Abstract

Splanchnetopia accompanied with atrial septal defect is a rare congenital malformation clinically. Recent studies show that the patients are susceptible to lung disease. We present a case of a 62-year-old man who presented to our hospital with cough, palpitation and short of breath after activity. On physical examination, we found cyanosis of lips and face, swollen jugular vein, bubble sounds at the lung bottom, irregular heart rhythm, the large liver under the left rib and the lower limbs edema. Laboratory studies revealed white blood cell 18.6 × 10^9^/L and neutrophils 73.9%. Electrocardiogram showed disappearance of P wave and substituted F wave with irregular R-R interval. Ultrasound cardiogram indicated that there was a 24 mm-long gap in the middle of the atrial septal. Dextrocardia, pulmonary artery extrudes and infection of lung were found by chest x-ray. The abdomen ultrasound indicated organ flip. Clinical diagnosis was congenital heart disease, splanchnectopia, atrial septal defect, Eisenmenger’s syndrome, atrial flutter, cardiac function class III and pneumonia. The patient left hospital after 2-week treatment. Its clinical significance is when the thoracic and celiac organs are ill, the position of the symptoms and physical signs are contrary to the normal place. Therefore, we should make a careful and systematic examination of the patients in order to avoid misdiagnosis and delay in treatment.

## Introduction

Congenital dextrocardia means the heart position in the chest moves to the right. Dextrocardia accompanied by completely splanchnic inversion is called as Splanchnectopia. Pure dextrocardia does not cause pathological and physiological changes nor any symptoms. It may also suffer from the acquired heart disease like normal person. Splanchnetopia accompanied with atrial septal defect is a rare congenital malformation clinically [1]. Report is as follows.

## Case Report

**Figure 1:**
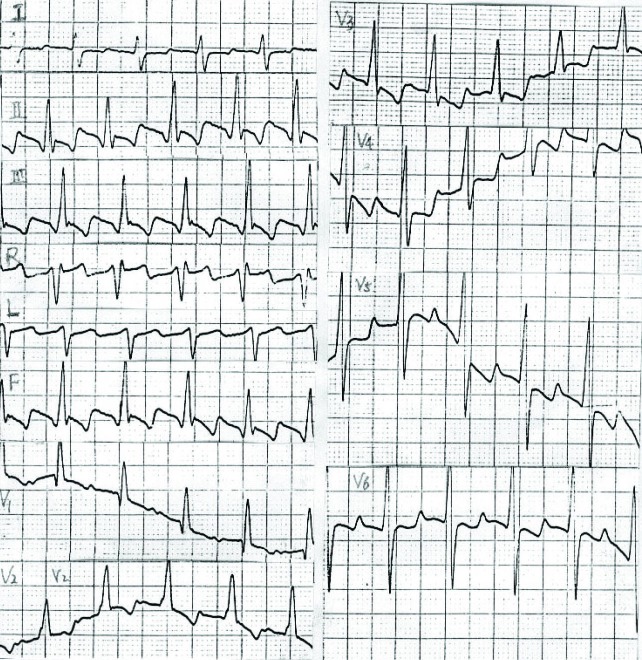
Electrocardiogram showing atrial flutter.

**Figure 2:**
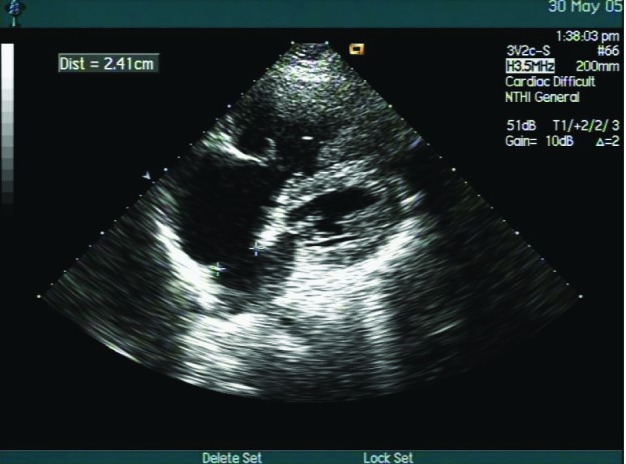
Ultrasound cardiogram showing the atrial septal defect.

A 62-year-old man was admitted to our hospital because of cough, palpitation and short of breath after activity. Physical examinations were done after admission. The blood pressure was measured 140/80 mmHg. Cyanosis of lips and face and swollen jugular vein were found. Some bubble sounds were heard at the base of the lung. The heart dullness was expanded slightly. The heart rate was 90 times/min and the rhythm was absolutely irregular. No murmurs at every valvular area were heard. The liver brim was palpated under the left rib and edema was seen in the lower limbs. Laboratory test showed white blood cell 18.6 × 10^9^/L and neutrophils 73.9%. Electrocardiogram showed disappearance of P wave and substituted F wave with irregular R-R interval. The QRS wave of the lead V_1_ was qR, Rv_1_ + Rv_5_ > 1.05 mv (Fig. 1). Ultrasound cardiogram indicated that the interval diameters were dilated in the right ventricle, left and right atrial, the aorta, left and right pulmonary artery. The front wall of the right ventricle became hypertrophy. There was a 24 mm-long gap in the middle of the atrial septal (Fig. 2). Doppler flow imaging demonstrated bi-direction flow in the atrial septal defect. Dextrocardia, pulmonary artery extrudes and infection of lung were found by chest x-ray. The abdomen ultrasound indicated organ flip. Clinical diagnosis was congenital heart disease, splanchnectopia, atrial septal defect, Eisenmenger’s syndrome, atrial flutter, cardiac function class III and pneumonia. The patient left hospital after 2-week treatment.

## Discussion

Congenital dextrocardia was first described by Maco Aurelio Severino in 1963 [2]. It is considered as the main part of heart is located in the right thoracic cavity, caused by the left bend of primitive cardiac tube in the 5th or 6th weeks in the early embryo. Dextrocardia is divided into three types by Van Praagh analyzing method [2, 3]: 1) mirror-image dextrocardia, often with splanchnectopia, including the right atrial and liver in the left, while left atrial and fundus of stomach in the right of spinal cord; the left three lobed and right two lobes of lung; the reversion of left and right bronchi. The case mentioned in this article belongs to this kind. 2) Dextroversion: the heart is located in the right thoracic cavity but the other organs are in normal place. 3) Isolated dextrocardia, a particular kind of dextrocardia: there is no viscera inversion, but the veins and atrial are located in the left of the spine as the mirror-image dextrocardia. The liver superior segment of the inferior cava suddenly turns to left from the right side of the spine into the atrial which is often accompanied with cardiovascular malformation. The incidence of the mirror-image dextrocardia is 0.01%, which was believed to be seldom intracardiac malformation in the past [4]. Recent studies show that malformation of the heart is up to 40% - 50% and the patients are readily accompanied by lung disease [5]. About 15% - 25% of the patients suffer from Kartagener’s Syndrome, a syndrome of dextrocardia with chronic rhinitis, bronchiectasis, rhinopolypus or male sterility. The main reason of the syndrome is the abnormal activity of the villus [6]. The case mentioned above is splanchnetopia accompanied with atrial septal defect. Its clinical significance is when the thoracic and celiac organs are ill, the position of the symptoms and physical signs are contrary to the normal place because of splanchnectopia. Therefore, we should make a careful and systematic examination of the patients in order to avoid misdiagnosis and delay in treatment.
